# A combined HM-PCR/SNuPE method for high sensitive detection of rare DNA methylation

**DOI:** 10.1186/1756-8935-3-12

**Published:** 2010-06-02

**Authors:** Sascha Tierling, Matthias Schuster, Reimo Tetzner, Jörn Walter

**Affiliations:** 1 Universität des Saarlandes, FR. 8.3 Biowissenschaften, Genetik/Epigenetik, 66041 Saarbrücken, Germany; 2 Dx assays, Blk 35 Marsiling Ind Estate Road 3 Woodlands Central Industrial Estate, 739257, Singapore; 3 Epigenomics AG, Kleine Präsidentenstr. 1, 10178 Berlin, Germany

## Abstract

**Background:**

DNA methylation changes are widely used as early molecular markers in cancer detection. Sensitive detection and classification of rare methylation changes in DNA extracted from circulating body fluids or complex tissue samples is crucial for the understanding of tumor etiology, clinical diagnosis and treatment. In this paper, we describe a combined method to monitor the presence of methylated tumor DNA in an excess of unmethylated background DNA of non-tumorous cells. The method combines heavy methyl-PCR, which favors preferential amplification of methylated marker sequence from bisulfite-treated DNA with a methylation-specific single nucleotide primer extension monitored by ion-pair, reversed-phase, high-performance liquid chromatography separation.

**Results:**

This combined method allows detection of 14 pg (that is, four to five genomic copies) of methylated chromosomal DNA in a 2000-fold excess (that is, 50 ng) of unmethylated chromosomal background, with an analytical sensitivity of > 90%. We outline a detailed protocol for the combined assay on two examples of known cancer markers (SEPT9 and TMEFF2) and discuss general aspects of assay design and data interpretation. Finally, we provide an application example for rapid testing on tumor methylation in plasma DNA derived from a small cohort of patients with colorectal cancer.

**Conclusion:**

The method allows unambiguous detection of rare DNA methylation, for example in body fluid or DNA isolates from cells or tissues, with very high sensitivity and accuracy. The application combines standard technologies and can easily be adapted to any target region of interest. It does not require costly reagents and can be used for routine screening of many samples.

## Background

Changes in DNA methylation such as hypermethylation of tumor suppressor genes are regarded as early molecular events during cancer development. Such epigenetic changes are widely used as molecular markers in tumor cell diagnostics [1-3]. Selective and robust PCR-based screening methods for very early detection of abnormal tumor-specific methylation become increasingly important. In particular, methods to screen for the low abundance of aberrantly methylated tumor DNA present in peripheral blood samples or other body fluids are regarded as very promising, non-invasive, early cancer detection tests [4].

**Figure 1:**
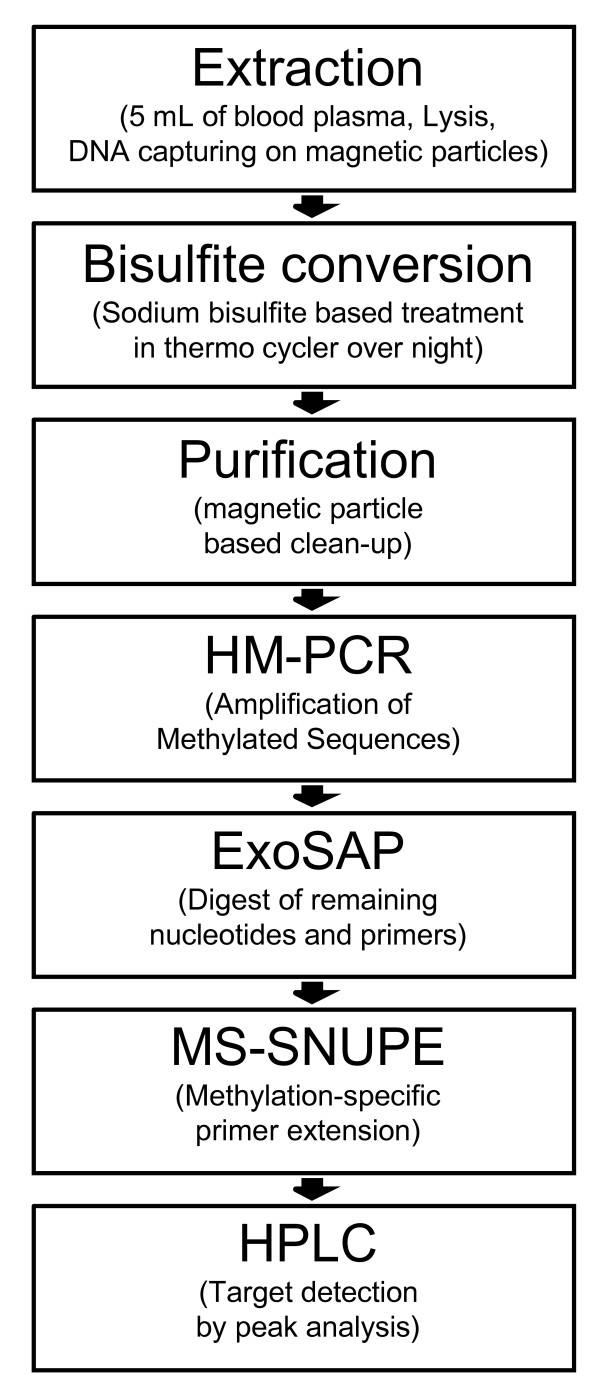
**Workflow of the combined heavy methyl (HM)-PCR/methylation-restricted single nucleotide primer extension (MR-SNuPE) assay**.

Although many potentially diagnostic DNA methylation markers for such sensitive tests have been identified for several solid tumor types (for example, colon, breast or prostate cancer) only a few have passed validation tests in larger clinical studies on blood plasma samples [5-9]. One reason not to gain faster progress in 'biomarker' validation is the lack of robust and versatile methods for comprehensive and robust clinical testing. A combination of real-time PCR using either methylation-specific primers (MS-PCR)[10] or blockers (heavy methyl-PCR; HM-PCR) [11] with methylation-specific fluorescence probes (for example, MethyLight) [12] have successfully been used to detect low copy numbers of aberrantly methylated tumor DNA in blood plasma samples [12,13]. In this paper, we present a novel assay combining sensitive HM-PCR with methylation-restricted single nucleotide primer extension (MR-SNuPE) followed by ion-pair, reversed-phase, high-performance liquid chromatography IIP/RP-HPLC) detection. The combined assay has the highest analytical sensitivity, allows an excellent product and quality monitoring, and is as sensitive as real-time PCR-based assays. Technically the assay is easy to perform, including semi-automatable processing steps (see workflow outlined in Figure 1). It requires a HPLC system but otherwise operates at relatively low cost, as it does not rely on the use of complex chemistry. We demonstrate the sensitivity and application of the method to routine diagnostics in a pilot study on two pre-validated colon cancer methylation markers, transmembrane epidermal growth factor (*TMEFF2/TPEF*) and septin 9 (*SEPT9*) [14-18].

## Results

**Figure 2:**
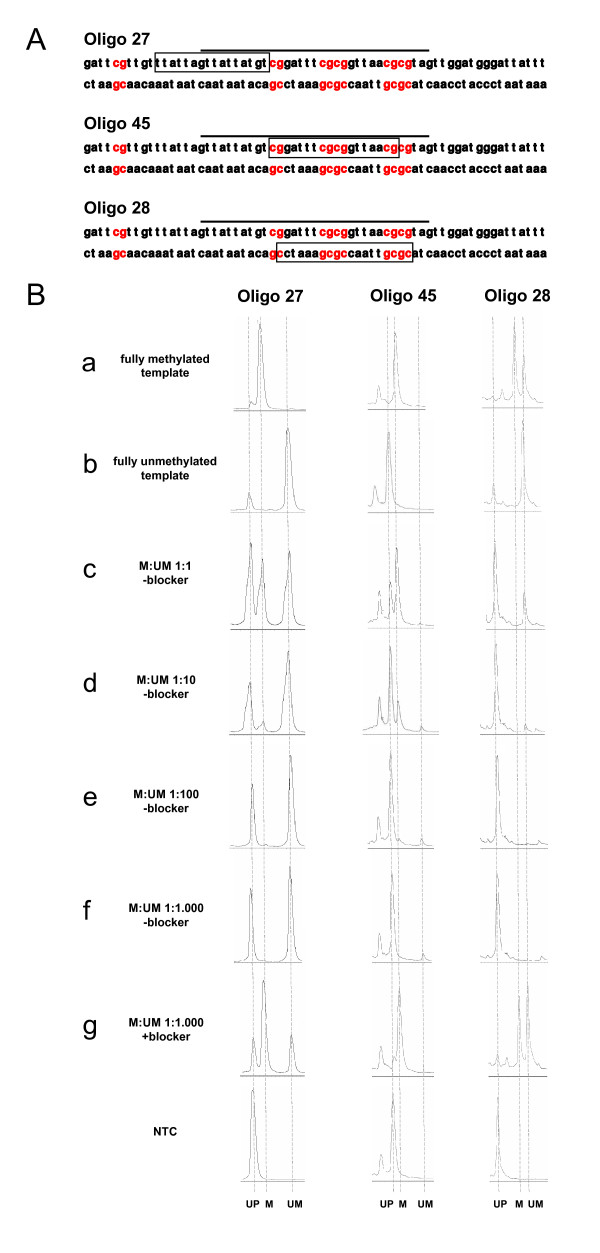
**MR-SNuPE assay design for *SEPT9 *and general performance**. **(A) ***SEPT9 *amplicon sequence with indicated primer (boxes) and blocker (line above) positions. **(B) **Electropherograms of separated single nucleotide primer extension (SNuPE) products from PCR products obtained without blocker on **(a) **completely methylated and **(b) **unmethylated DNA templates or **(c-f) **mixed DNA templates (methylated:unmethylated DNA ratios/dilution series). **(g) **Effect when performing a heavy methyl (HM)-PCR; that is, when the blocker is included, on a dilution shown in **(f)**. Vertical dashed lines indicate the positions of unextended primer, methylated and unmethylated signals, respectively, NTC = no template control, that is, SNuPE reaction without PCR template.

We first determined the limits of SNuPE detection by IP/RP-HPLC on PCR products obtained by conventional PCR of bisulfite-treated template DNA. We mixed decreasing amounts of fully methylated template (1:1 to 1:1,000) in an excess of fully unmethylated bisulfite-treated DNAs. These mixed template DNAs were treated with bisulfite and then used to generate *SEPT9*- and *TMEFF2*-specific PCR products. Amplicons were subsequently treated with an exonuclease I/shrimp alkaline phosphatase (SAP) mix to remove PCR primers and dephosphorylate dNTPs, followed by heat inactivation and direct use of CpG-specific primer extension (see workflow Figure 1). Primers were designed for both strands of the PCR products, and extension reactions were performed with regular unlabelled ddNTPs. For extension primers we used either the CpG-free (unbiased recognition of bisulfite DNA; MS-SNuPE)[19] primers 'oligo 27' for *SEPT9 *and 'oligo 30' for *TMEFF2*, or the (methylation-restricted; MR-SNuPE) CpG dinucleotide-containing primers 'oligo 45' for *SEPT9 *and 'oligo 62' for *TMEFF2 *(top strand) and 'oligo 28' for *SEPT9 *and 'oligo 44' for *TMEFF2 *(bottom strand) (Figure 2A; see Additional file 1, supplemental figure 1A). SNuPE reaction products were individually separated on a column (DNASep™ Cartridge; Transgenomic, Omaha, NE, USA) using a DNA fragment analyzer (WAVE™ DNA Fragment Analysis System; Transgenomic). Figure 2B shows the MS-SNuPE and MR-SNuPE electropherograms obtained for the *SEPT9*-specific extensions (primers 27, 28 and 45). Product peaks (ultraviolet spectra at 260 nm) representing methylated and unmethylated templates (designated M and UM in the figures) were identified on fully methylated and unmethylated amplicons, respectively (Figure 2B (a and b)). We recommend performing such control reactions to standardize experimental series because absolute retention times can change depending on the age of the HPLC column. Moreover, a no-template control (NTC) was carried along with each MS-SNuPE and MR-SNuPE reaction to reveal the retention time of the unextended primer and unspecific primer products (for example, see oligo 45 in Figure 2B). The incremental dilutions of methylated templates in an unmethylated background revealed small but reproducibly detectable methylation-specific primer extensions even in mixtures containing only one methylated copy in 100 unmethylated copies (that is, a detection limit of about 1%). Although the detection of methylation was robust for all primers, the presence of unmethylated products obviously influences the sensitivity and specificity of the reaction. For example, unmethylated extension products were obtained with oligo 28 and oligo 45, which should only bind to and extend methylated templates (Figure 2B). Analogous results were obtained for *TMEFF2*-specific reactions (see Additional file 1, supplemental figure 1B). To suppress unspecific extension and enhance the detection sensitivity, a PCR blocker (for position within the PCR products see Figure 2A, Additional file 1, supplemental figure 1A) which specifically binds and suppresses the amplification of unmethylated templates, was included in the PCR reactions. The presence of this blocker significantly enhances the enrichment of methylated products. Clear methylation-specific extension products (designated as M; Figure 2B (g); see Additional file 1, supplemental figure 1A (g)) were visible down to a 1:1,000 dilution. Nevertheless, the blocker does not completely suppress the amplification of unmethylated templates, which could be visualized as primer extension products when using the oligos 27, 30, 62, 44 and 28, which produce unmethylated extension products (UM in the figure). Note that the signals representing unmethylated products for oligo 28 were at least partially caused by its complementarity to the blocker that is present in the reaction, hence, the presence of the blocker should be taken into account when performing primer design.

In summary, the analysis performed on two amplicons revealed that MR-SNuPE reactions performed on templates generated by conventional unbiased PCR already reach high levels of detection, that is, at least one methylated copy could easily and unambiguously be detected in a background of 1,000 unmethylated copies. However, in MR-SNuPE reactions, detection of co-methylated templates is favored because oligos are used that include CpGs (oligos 28 and 45; oligos 44 and 62). Extension primers without CpGs (oligos 27 and 30) bind independently of the methylation state (MS-SNuPE reaction) and might not be as sensitive but allow simultaneous detection of co-amplified non-methylated templates and, hence, serve as a good HM-PCR (or MS-PCR) quality control (see below).

To enhance the sensitivity we then combined SNuPE reactions on PCR products produced by HM-PCR. To define the limit of detection in such double selective reactions for very low DNA concentrations such as in blood plasma DNA samples, we again performed a proof of principle experiment. We spiked defined amounts of methylated templates (6.25, 12.5, 25, 50 and 100 pg) into unmethylated background (50 ng) and performed primer extension reactions on 10 to 24 HM-PCR reactions for each 'dilution'. For *SEPT9 *we observed a 100% detection down to 25 pg of spiked methylated template (corresponding to 1 methylated copy in 500 to 2,000 unmethylated copies) (see Additional file 1, supplemental Figure 2A, supplemental Figure 2B and supplemental table 1). A detection rate of 83.3% was obtained with 12.5 pg (1 in 4,000) and 58.3% with 6.25 pg (1 in 8,000) methylated template. Hence, with a 90% probability (Probit analysis), the method allows detection of 13.6 pg (CI 9.6 to 193 pg) of methylated copies in an excess of unmethylated background for *SEPT9 *and 21.9 pg (CI 13.0 to 36.9 pg) for *TMEFF2 *(Additional file 1, supplemental Figure 2C, supplemental Figure 2D). Hence, the combined HM-PCR/MR-SNuPE assay reaches sensitivities close to the theoretical optimum.

**Figure 3:**
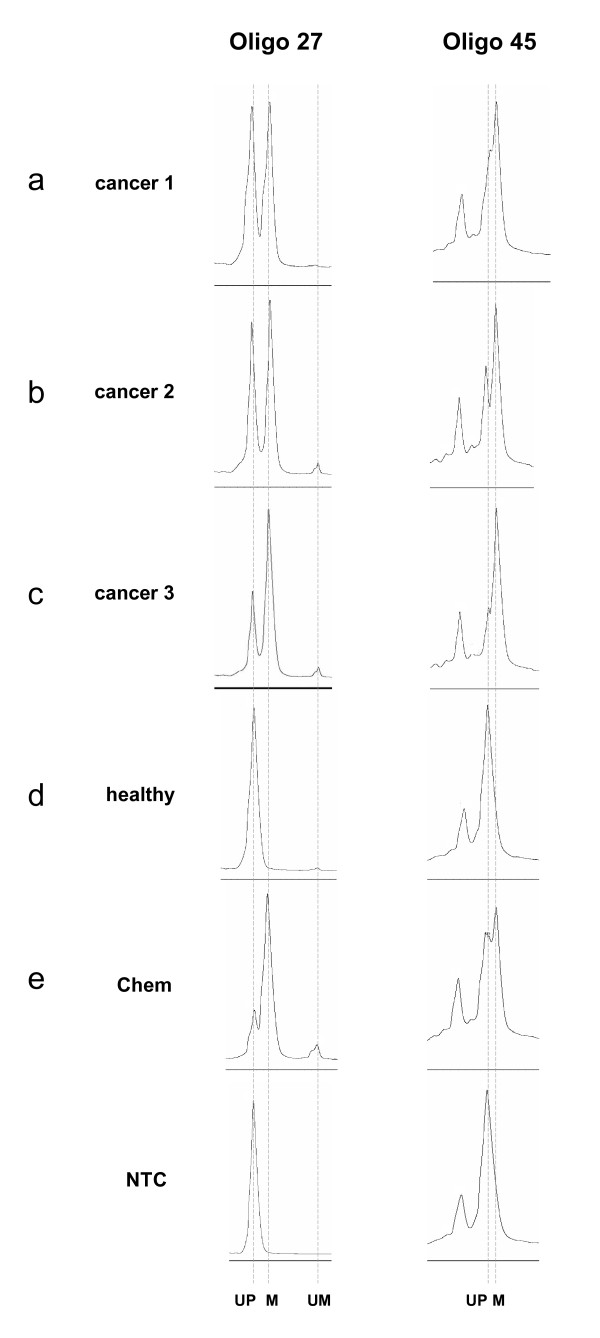
**Assay performance tested on samples from patients with colorectal cancer**. Electropherograms after separation of methylation-restricted single nucleotide primer extension (MR-SNuPE) products obtained from plasma taken from patients with colonoscopy-verified colorectal cancer. Plasma from **(a-c) **patient with cancer, obtained from Proteogenex (PRO6, PRO20) or Oncomatrix (OMA19); **(d) **healthy individual, obtained from Oncomatrix (OMA8). **(e) **Normal blood plasma (5 ml) spiked with 12.5 ng methylated DNA (Chem). Peaks were assessed by the relative signal retention times as described in the legend to Figure 2. UP = unextended primer, M = methylated signal; NTC = SNuPE reaction with water.

In a double-blind study, we then used the combined HM-PCR/MR-SNuPE assay to detect methylation at *SEPT9 *in free-floating tumor DNA isolated from blood plasma of colon cancer probands. One-fifth of bisulfite-treated DNA samples isolated from 4-5 ml of plasma taken from 20 patients with colorectal cancer (CRC) and 20 healthy donors was used for HM-PCR and subsequent MS-SNuPE and MR-SNuPE. In all 20 healthy donor samples, only unmethylated products were seen, whereas in the CRC samples, methylated products were found in 10 of the 20 cases by HM-PCR/primer extension assays using oligo 27 and oligo 45 (Figure 3). In a real-time coupled HM-PCR assay, 11 of the 20 CRC samples were found to be methylation-positive (data not shown) [11]. Both methods together detected 13 of 20 cases as methylation-positive, but were discordant for five samples: two cases were clearly detected by HM-PCR/MR-SNuPE and not by real-time HM-PCR alone, and the other three cases were only positive by real-time HM-PCR. When analyzing the threshold cycles (CT) of the corresponding real-time PCRs, we observed that methylated DNA detection was in all cases close to the single copy level in the respective samples (data not shown). The discordant detection of five cases is therefore most likely a result of stochastic PCR variability on extremely low concentrations of methylated DNA in the respective plasma samples. In fact, both the real-time HM-PCR and the HM-PCR/SNuPE assays were performed on different aliquots of the same bisulfite-treated DNA. Thus, we recommend performing assays in replicates to decrease the number of false negatives resulting from limited amounts of methylated copies. Overall, our pilot experiment shows that there is no significant difference in the performance and sensitivity of both methods (a McNemar test for both methods on the 20 CRC samples showed a similar performance with *P *= 1.0).

## Discussion

By combining two highly selective DNA methylation enrichment techniques (HM-PCR and MR-SNuPE), we generated a novel assay with extremely sensitive detection rates. With this combination, we were able to detect 14 pg methylated template DNA (~ four to five haploid copies) in 50 ng of unmethylated DNA. The method reached a detection level close to the theoretical limit. We then performed a small pilot study on plasma DNA samples from 20 patients with CRC and 20 healthy individuals to demonstrate that the method can in principle be used for routine analysis in biomedical research.

All of the main assays such MS-PCR, MethyLight or HM-PCR used for the detection of rare methylation in DNA samples are based on selective bisulfite-PCR amplification, but differ in the manner of data readout and the level of sensitivity [20-23]. In comparison to the real-time-based methods, MethylLight and other HM-PCR-based assays, the combined HM-PCR/MR-SNuPE is a simple end-point analysis that is comparable with MS-PCR but is more sensitive and selective. It does not require sophisticated chemistry but still achieves an excellent detection level. Moreover, by using combinations of MS-SNuPE and MR-SNuPE primers, it is possible to assess several CpG positions independently and also to quality control the selective methylation-specific PCR performance (see below). Very recently, another alternative assay reaching a similar sensitivity was reported. That method uses heat-stable restriction enzymes during PCR [24]. By contrast, our method is not limited to the presence of distinct restriction sites in the amplicon of interest and, hence, is more flexible in its application. A further advantage is that it does not require extensive purification steps or complex and costly nucleotide chemistry for the analysis.

In our experience, the combination of SNuPE assays with HM-PCR assays (and presumably MS-PCR also) requires very little HPLC optimization. However, great care should be taken with primer design for both MS-SNuPE and MR-SNuPE reactions. We recommend using 12-16 nucleotides long HPLC-purified oligos. To achieve the highest selectivity and sensitivity in MR-SNuPE reactions, the oligos should encompass at least three CpG dinucleotides. In addition, any complementarity with the blocker used in HM-PCR should be avoided. For optimum HPLC separation, primers should be generated from the A-rich strand to generate either ddCTP or ddTTP extensions. Although the number of methylation-specific CpG positions in the extension oligos (such as oligo 45 that includes four CpGs) enhances the specificity for methylated products only, the detection sensitivity is equally good with oligos that additionally detect co-amplified non-methylated templates in the HM-PCR reaction (see oligo 27 in Figure 2B (g), for example). We recommend including at least one unbiased (MS-SNuPE) extension primer (oligos 27 and 30 in our example) in the assay. This allows simultaneous detection of unmethylated and methylated products, serves as a direct quality control for the HM-PCR (MS-PCR) amplification, and monitors the accuracy of the SNuPE extension. Besides the high sensitivity and specificity, the combination of HM-PCR and SNuPE detection has several practical advantages for routine sensitive diagnostics. First, the inclusion of a blocker for unmethylated DNA into the HM-PCR reaction significantly reduces the amount of unmethylated byproducts and enhances the sensitivity and specificity of the SNuPE detection. A similar principle is used in MS-PCR assays when primers are placed across several methylated CpG positions [10]. In contrast to MS-PCR, the use of unbiased primers and the presence of selective blockers in our assay reduce but do not eliminate the amplification of unmethylated DNA. Moreover, the assay does not require complete co-methylation of all CpG positions. Secondly, our approach represents an all-or-nothing detection, that is, methylated DNA templates (above the detection threshold) always provide strong signals/peaks so that misinterpretations (false positives) can almost invariably be excluded.

Because the detection limit may easily be reached in plasma DNA samples (as seen in our small study) and because individual DNA samples might stochastically vary in the amount of tumor DNA present, the problem of false negatives has to be considered. We therefore suggest performing reactions in triplicate and, if possible, on independent DNA isolates and on several genes/amplicons. We are convinced that this robust combined HM-PCR/MR-SNuPE assay will be a useful diagnostic tool for the fast and cost-efficient detection of rare DNA methylation and will be applicable to larger cohort studies.

## Conclusions

The described HM-PCR/MR-SNuPE assay is a robust and versatile endpoint analysis method for high sensitive detection of rare DNA methylation in complex DNA samples. The assay is cost-efficient and allows semi-automated processing of samples, making it applicable for routine testing in DNA samples isolated from biopsy tissues or body fluids.

## Methods

### DNA and sample material

Universal *in vitro *methylated human DNA was obtained from Millipore. Unmethylated DNA was prepared by molecular displacement amplification (MDA)[25] of 10 ng peripheral blood leukocyte DNA (Promega, Mannheim, Germany) using a commercial kit (Repli-G Kit; Qiagen, Hilden, Germany). Blood plasma from 20 patients with CRC (stages I, II, III) and 20 colonoscopy-verified normal controls was obtained from Oncomatrix and ProteoGenex, where samples were collected after informed consent and in compliance with local guidelines.

### Sample preparation

DNA was isolated from 5 ml of blood plasma and bisulfite converted [26] using previously established procedures (for detailed information see Additional file 1).

### Real-time PCR

HM-PCR was performed in 96-well plates on a thermal cycler (LightCycler 480; Roche, Mannheim, Germany). For both *SEPT9 *and *TMEFF2*, each 25 μl reaction contained 300 nM each of both forward and reverse primers (Table 1), 1 μM blocker and 100 nM of probe in 1× buffer (QuantiTect mPCR Kit NoROX; Qiagen, Hilden, Germany). After 30 minutes at 95°C, 50 cycles of PCR were performed: 30 seconds at 56°C and 10 seconds at 95°C for *SEPT9; *30 seconds at 56°C, 10 seconds at 72°C, 10 seconds at 95°C for *TMEFF2*. The mixture was then cooled to 40°C.

**Table 1:** Oligos used in the assays

Gene name	Oligo name	Sequence 5'→3'
*SEPT9*	Forward	GATT-X-GTTGTTTATTAGTTATTATGT

	Reverse	AAATAATCCCATCCAACTA

	Blocker	GTTATTATGTTGGATTTTGTGGTTAATGTGTAG-C3

	Probe	FAM-TTAACCGCGAAATCCGAC-BHQ1

	Oligo 27	ttattagttattatgt

	Oligo 28	cggatttcgcggttaacg

	Oligo 45	cgcgttaaccgcgaaatcc

*TMEFF2*	Forward	GGTTATTGTTTGGGTTAATAAATG

	Reverse	AAAAAAAAAAAACTCCTCTACATAC

	Blocker	ACATACACCACAAATAAATTACCAAAAACATCAACAA-C3

	Probe	FAM-TTCGGACGTCGTTGTTCGG-BHQ1

	Oligo 30	gttaataaatggagtt

	Oligo 44	gaacaacgacgtcc

	Oligo 62	cgtcgttgttcggt

X = tetrahydrofuran-type abasic site.

### HM-PCR, primer extension and HPLC separation

For methylation analysis by SNuPE, HM-PCR was performed as described above, omitting the probe. An aliquot (5 μl) of the HM-PCR product were treated with 1 μl of Exo-SAP (1:10 mixture of exonuclease I and SAP (USB, Staufen, Germany) for 30 min at 37°C. To inactivate the Exo-SAP enzymes, the reaction was incubated for 15 minutes at 80°C. Primer extension was carried out as described previously[27].

For *SEPT9*, to the PCR product/Exo-SAP mix, 2 μl of 10× buffer C (Solis BioDyne), 2.4 μl of 30 μM SNuPE primer (oligos 27, 28 and 45), 1 μl of 1 mM ddCTP and ddTTP or ddGTP and ddATP, respectively, and 0.5 μl of Termipol DNA polymerase (5 U/μl, Solis BioDyne) were added to reach a final volume of 20 μl. Reactions were performed in a thermal cycler under the following conditions: 96°C for 2 minutes, followed by 50 cycles 96°C for 30 seconds, 50°C for 30 seconds and 60°C for 2 minutes. Separation of SNuPE products was conducted at 50°C by continuously mixing buffer B (0.1 M triethylammonium acetate (TEAA), 25% acetonitrile) to buffer A (0.1 M TEAA) over 10 minutes, resulting in a buffer B concentration of 28% to 35% for oligo 27, 20% to 27% for oligo 28, and 21% to 27% for oligo 45.

For *TMEFF2*, To the PCR product. Exo-SAP mix, 2 μl of 10× buffer C (Solis BioDyne), 2.4 μl of 30 μM SNuPE primer (oligos 30, 44 and 62), 1 μl of 1 mM ddCTP and ddTTP or ddGTP and ddATP, respectively, and 0.5 μl of Termipol (5 U/μl; Solis BioDyne) were added to reach a final volume of 20 μl. Reactions were performed in a thermal cycler under the following conditions: 96°C for 2 minutes followed by 50 cycles of 96°C for 30 seconds, 60°C for 30 seconds and 60°C for 2 minutes. Separation of SNuPE products was conducted at 50°C by continuously mixing buffer B (0.1 M TEAA, 25% acetonitril) to buffer A (0.1 M TEAA) over 10 minutes, resulting in a buffer B concentration of 21% to 29% for oligo 30, 16% to 26% for oligo 44, and 18% to 28% for oligo 62.

### Cloning and bisulfite sequencing of methylated and unmethylated *SEPT9 *amplicons

For preparation of methylated and unmethylated *SEPT9 *amplicons, 5 ng of bisulfite-treated methylated and unmethylated DNA, respectively, were amplified by HM-PCR without using a probe. PCR products were cloned into pGemT vector (Promega, Mannheim, Germany) according to the manufacturer's protocol, and transformed into *Escherichia coli *TOP10 cells. Positive clones were checked by colony PCR and subsequently sequenced. Complete unmethylated and methylated clones were used for HPLC separation.

### Limit of detection

In total, 50 ng of bisulfite-treated unmethylated DNA was spiked with subnanogram amounts (100, 50, 25, 12.5, 6.25, 0 pg) of bisulfite-treated methylated DNA and analyzed in replicate by both methods in parallel. The limit of detection was defined as the minimum amount of bisulfite-treated methylated DNA that could be distinguished from unmethylated background with 90% confidence.

## Competing interests

JW is a cofounder of Epigenomics AG, Berlin and works as a scientific advisor for the company. RT is employee and shareholder of the Epigenomics AG. SEPT9 is a diagnostic biomarker commercialized by Epigenomics AG. ST and MS declare no conflict of interest.

## Authors' contributions

ST, MS, RT and JW made substantial contributions to conception and design of the study. ST designed and performed the SNuPE experiments. MS and RT designed and performed HM-PCR experiments. All authors read and approved the final manuscript.

## Supplementary Material

Additional file 1 **Supplementary material**. Click here for file

## References

[B1] Ozanne SE Constancia M Mechanisms of disease: the developmental origins of disease and the role of the epigenotype Nat Clin Pract Endocrinol Metab 2007 3 539 546 10.1038/ncpendmet0531 17581623

[B2] Gronbaek K Hother C Jones PA Epigenetic changes in cancer APMIS 2007 115 1039 1059 10.1111/j.1600-0463.2007.apm_636.xml.x 18042143

[B3] Esteller M Epigenetic gene silencing in cancer: the DNA hypermethylome Hum Mol Genet 2007 16 R50 59 10.1093/hmg/ddm018 17613547

[B4] Chatterjee SK Zetter BR Cancer biomarkers: knowing the present and predicting the future Future Oncol 2005 1 37 50 10.1517/14796694.1.1.37 16555974

[B5] Herman JG Hypermethylation pathways to colorectal cancer. Implications for prevention and detection Gastroenterol Clin North Am 2002 31 945 958 10.1016/S0889-8553(02)00058-4 12489271

[B6] Gonzalgo ML Liang G Spruck CH Zingg JM Rideout WM Jones PA Identification and characterization of differentially methylated regions of genomic DNA by methylation-sensitive arbitrarily primed PCR Cancer Res 1997 57 594 599 9044832

[B7] Toyota M Ho C Ahuja N Jair KW Li Q Ohe-Toyota M Baylin SB Issa JP Identification of differentially methylated sequences in colorectal cancer by methylated CpG island amplification Cancer Res 1999 59 2307 2312 10344734

[B8] Yan PS Efferth T Chen HL Lin J Rödel F Fuzesi L Huang TH Use of CpG island microarrays to identify colorectal tumors with a high degree of concurrent methylation Methods 2002 27 162 169 10.1016/S1046-2023(02)00070-1 12095276

[B9] Model F Osborn N Ahlquist D Gruetzmann R Molnar B Sipos F Galamb O Pilarsky C Saeger HD Tulassay Z Hale K Mooney S Lograsso J Adorjan P Lesche R Dessauer A Kleiber J Porstmann B Sledziewski A Lofton-Day C Identification and validation of colorectal neoplasia-specific methylation markers for accurate classification of disease Mol Cancer Res 2007 5 153 163 10.1158/1541-7786.MCR-06-0034 17314273

[B10] Herman JG Graff JR Myohänen S Nelkin BD Baylin SB Methylation-specific PCR: a novel PCR assay for methylation status of CpG islands Proc Natl Acad Sci USA 1996 93 9821 9826 10.1073/pnas.93.18.9821 8790415 PMC38513

[B11] Cottrell SE Distler J Goodman NS Mooney SH Kluth A Olek A Schwope I Tetzner R Ziebarth H Berlin K A real-time PCR assay for DNA-methylation using methylation-specific blockers Nucleic Acids Res 2004 32 e10 10.1093/nar/gnh008 14722226 PMC373310

[B12] Eads CA Danenberg KD Kawakami K Saltz LB Blake C Shibata D Danenberg PV Laird PW MethyLight: a high-throughput assay to measure DNA methylation Nucleic Acids Res 2000 28 e32 10.1093/nar/28.8.e32 10734209 PMC102836

[B13] Cottrell SE Laird PW Sensitive detection of DNA methylation Ann N Y Acad Sci 2003 983 120 130 10.1111/j.1749-6632.2003.tb05967.x 12724217

[B14] Liang G Robertson KD Talmadge C Sumegi J Jones PA The gene for a novel transmembrane protein containing epidermal growth factor and follistatin domains is frequently hypermethylated in human tumor cells Cancer Res 2000 60 4907 4912 10987305

[B15] Sabbioni S Miotto E Veronese A Satin E Gramantieri L Bolondi L Calin GA Gafà R Lanza G Carli G Terrazzi E Feo C Liboni A Rullini S Negrini M Multigene methylation analysis of gastrointestinal tumors: TPEF emerges as a frequent tumor-specific aberrantly methylated marker that can be detected in peripheral blood Mol Diagn 2003 7 201 207 10.2165/00066982-200307030-00010 15068392

[B16] Grützmann R Molnar B Pilarsky C Habermann JK Schlag PM Saeger HD Miehlke S Stolz T Model F Roblick UJ Bruch HP Koch R Liebenberg V Devos T Song X Day RH Sledziewski AZ Lofton-Day C Sensitive detection of colorectal cancer in peripheral blood by septin 9 DNA methylation assay PLoS One 2008 3 e3759 10.1371/journal.pone.0003759 19018278 PMC2582436

[B17] Lofton-Day C Model F Devos T Tetzner R Distler J Schuster M Song X Lesche R Liebenberg V Ebert M Molnar B Grützmann R Pilarsky C Sledziewski A DNA methylation biomarkers for blood-based colorectal cancer screening Clin Chem 2007 54 414 423 10.1373/clinchem.2007.095992 18089654

[B18] DeVos T Tetzner R Model F Weiss G Schuster M Distler J Vaughn-Steiger K Grützmann R Pilarsky C Habermann JK Day R Sledziewski A Lofton-Day C Circulating methylated septin 9 in plasma is a biomarker for colorectal cancer Clin Chem 2009 55 1337 1346 10.1373/clinchem.2008.115808 19406918

[B19] Gonzalgo ML Jones PA Rapid quantitation of methylation differences at specific sites using methylation-sensitive single nucleotide primer extension (Ms-SNuPE) Nucleic Acids Res 1997 25 2529 2531 10.1093/nar/25.12.2529 9171109 PMC146734

[B20] Xiong Z Laird PW COBRA - a sensitive and quantitative DNA methylation assay Nucleic Acids Res 1997 25 2532 2534 10.1093/nar/25.12.2532 9171110 PMC146738

[B21] Weber M Davies JJ Wittig D Oakeley EJ Haase M Lam WL Schübeler D Chromosome-wide and promoter-specific analyses identify sites of differential DNA methylation in normal and transformed human cells Nat Genet 2005 37 853 862 10.1038/ng1598 16007088

[B22] Frommer M McDonald LE Millar DS Collis CM Watt F Grigg GW Molloy PL Paul CL A genomic sequencing protocol that yields a positive display of 5-methylcytosine residues in individual DNA strands Proc Natl Acad Sci USA 1992 89 1827 1831 10.1073/pnas.89.5.1827 1542678 PMC48546

[B23] Wolf SF Migeon BR Studies of X chromosome DNA methylation in normal human cells Nature 1982 295 667 671 10.1038/295667a0 6173772

[B24] Kneip C Schmidt B Fleischhacker M Seegebarth A Lewin J Flemming N Seemann S Schlegel T Witt C Liebenberg V Dietrich D A novel method for sensitive and specific detection of DNA methylation biomarkers based on DNA restriction during PCR cycling Biotechniques 2009 47 737 744 10.2144/000113208 19852759

[B25] Dean FB Hosono S Fang L Wu X Faruqi AF Bray-Ward P Sun Z Zong Q Du Y Du J Driscoll M Song W Kingsmore SF Egholm M Lasken RS Comprehensive human genome amplification using multiple displacement amplification Proc Natl Acad Sci USA 2002 99 5261 5266 10.1073/pnas.082089499 11959976 PMC122757

[B26] Tetzner R Dietrich D Distler J Control of carry-over contamination of PCR-based DNA methylation quantification using bisulfite treated DNA Nucleic Acids Res 2007 35 e4 10.1093/nar/gkl955 17135186 PMC1747185

[B27] El-Maarri O SIRPH analysis: SNuPE with IP-RP-HPLC for quantitative measurements of DNA methylation at specific CpG sites Methods Mol Biol 2004 287 195 205 15273413 10.1385/1-59259-828-5:195

