# Supplementary material for: A combined HM-PCR/SNuPE method for high sensitive detection of rare DNA methylation
Source: Epigenetics Chromatin. 2010 Jun 2;3:12. doi: 10.1186/1756-8935-3-12 (PMC2887863; doi:10.1186/1756-8935-3-12)
Supplement: Additional file 1 — Supplementary material. [file 1756-8935-3-12-S1.DOC]

**Supplemental Material**

# Sample Preparation

DNA was isolated from 5 ml of blood plasma using **components of the Plasma DNA Extraction kit (Epigenomics,** M3-01-003). After transferring the plasma into 50ml Falcon tubes 5ml PDE Buffer1, 7µl PDE carrier and 30µl Proteinase K was added. Tubes were thoroughly vortexed for 10sec and incubated at 56°C for 10min. After addition of 100µl PDE beads and 15ml PDE Buffer 2 the tubes were briefly vortexed and subsequently mixed for 60 min using a rotating shaker. A magnetic separator was applied to retain the beads wheareas the buffer was removed by decanting and pipetting off any residuals. Subsequently 1.5ml wash PDE Buffer 3 was applied to completely resuspend the beads by pipetting up and down. Suspensions were transferred to 2ml Eppendorf tubes which were placed into the magnetic separator to remove the buffer. After a quick spin and removal of residual traces of buffer 100µl of PDE buffer 4 was added and tubes were placed in a thermoshaker at 65°C for 1400rpm. Tubes were again placed into the magnetic separator before eluates were carefully transferred to 0.5ml tubes for bisulfite conversion. To each eluate 190µl of Bisulfite Reagent and 30µl of Bisulfite Buffer DME containing 3.76mg 6-hydroxyl-2,5,7,8-tetramethylchroman-2-carboxylic acid (both part of the Bisulfite Conversion kit, Epigenomics, M3-01-004) were added and tubes were incubated in an Eppendorf PCR cycler at 99°C/5min, 50°C/25min, 99°C/5min, 50°C/85min, 99°C/5min, 50°C/295min and 20°C until removal from the block. Bisulfite DNA was purified using components of the Bisulfite DNA Purification kit (Epigenomics, M3-01-006). After removal from the PCR block reaction mixtures were transferred into 2ml Eppendorf tubes and 1µl BP Carrier, 1.5ml BP Buffer 1 and 10µl BP beads was added. Suspensions were vortexed briefly before incubation at 25°C in a thermoshaker at 1000rpm for 60min. Tubes were then placed into the magnetic separator and the buffer was removed completely. 300µl BP Buffer 2 was added, beads were carefully suspended by pipetting up and down and using the magnetic separator the BP Buffer 2 was removed. After one repetition of this wash step, one wash with 300µl BP Buffer 3 was performed using the same procedure. After complete removal of the BP Buffer 3 tubes were placed open in a thermoblock at 55°C for 5min. For elution 55µl BP Buffer 4 was added, the mixture was gently suspended and placed into a thermoshaker at 55°C and 1000rpm for 15min. After a quick spin tubes were again placed into the magnetic separator and eluates were carefully transferred into designated wells of a 96well plate for storage and further use. Bisulfite DNA prepared according to the described procedure is not desulfonated and thus represents SafeBis-DNA [24].

**Supplemental Figure 1: MR-SNuPE assay design for *TMEFF2* and performance**

**A** *TMEFF2* amplicon sequence with indicated primer (boxes) and blocker (line above) positions, **B** Electropherograms of separated SNuPE products from PCR products obtained without blocker on (a) completely methylated and (b) unmethylated DNA templates or (c-e) mixed DNA templates (methylated:unmethylated DNA ratios/dilution series). (f) shows the effect when performing an HM-PCR, i.e. when the blocker is included. Vertical dashed lines indicate the positions of unextended primer, methylated and unmethylated signals, respectively; NTC - SNuPE reaction without PCR template.

**Supplemental Figure 2**: Determination of the Limit of Detection (LOD) of the real time HM- PCR and combined HM-PCR/MR-SNuPE assays for *SEPT9* (A and B) and *TMEFF2* (C and D) and Probit analyses were performed on data received from assay repetitions on different concentrations of methylated template DNA. Each reaction contained 50 ng of non-methylated bisulfite-converted human DNA as background, whereas only 100, 50, 25, 12.5 or 6.25 picogram methylated human DNA was added. The 10 replicates with 100 pg and 50 pg were positive in all cases. Detection rate was between 55 – 100% for reactions with 6.25 – 25 pg (20-24 replicates) starting concentration. No signal was observed in any reaction without methylated DNA controls, which were performed in at least 16 replicates. Statistical analysis and graphics were generated using appropriate script for R (www.r-project.org/).

1. ***SEPT9* real time HM assay**


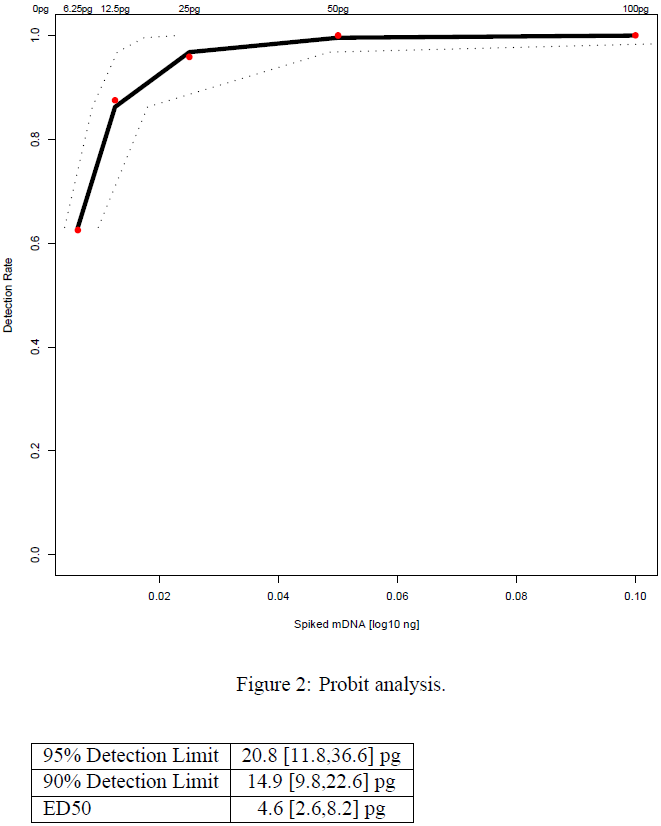


B) ***SEPT9* HM-PCR/MR-SNUPE**


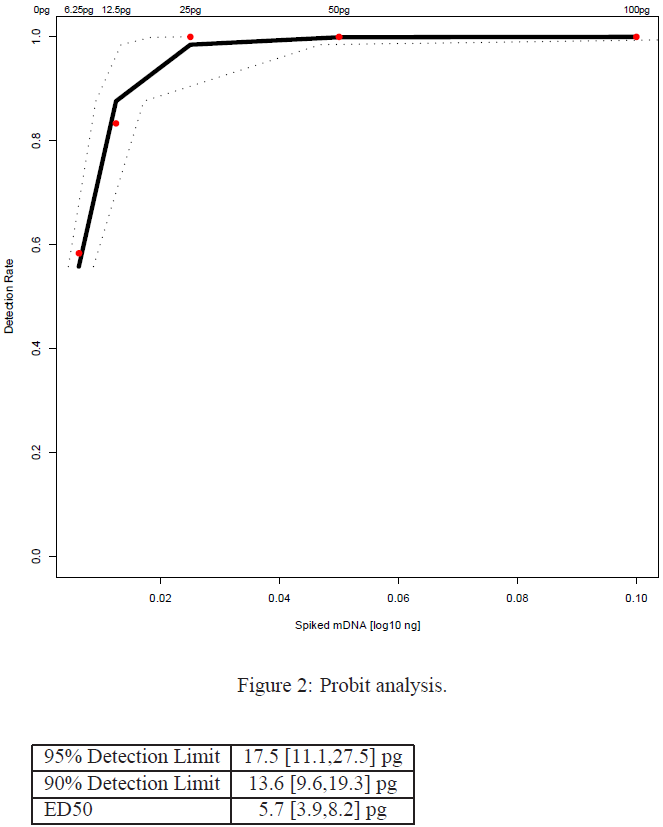


1. ***TMEFF2* real time HM assay**


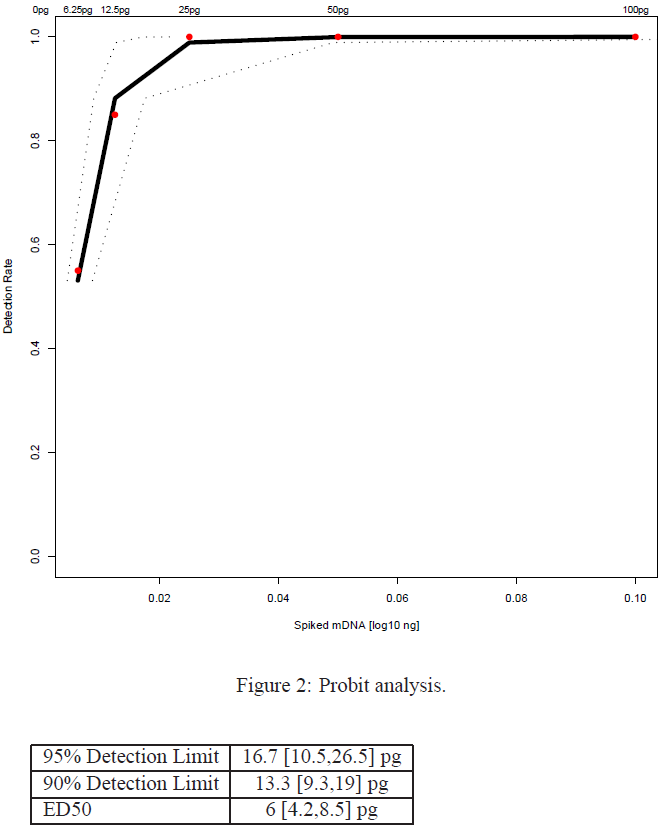


1. ***TMEFF2* HM-PCR/MR-SNUPE**


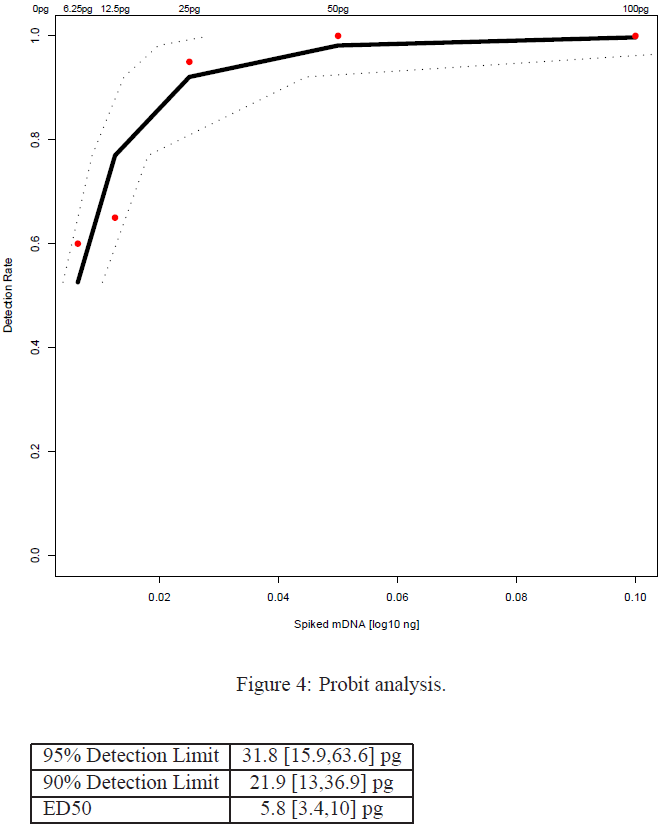


**Supplemental Table 1:** *SEPT9* level of detection (LOD) experiments using different incremental ratios of fully methylated Chem DNA spiked in 50 ng unmethylated background (UM) prior to HM-PCR. Primer extension reactions were performed on 10 and 24 different HM-PCR reactions per spike level, repectively. As controls 50 ng fully unmethylated DNA and a NTC were analyzed.

| **template mix** | **no.of reactions** | **used SNuPE primer** | **no.of methylated signals** | **% of methylated signals** |
| --- | --- | --- | --- | --- |
| NTC | 4 | p27 | 0 | 0 |
|  | 4 | p45 | 0 | 0 |
| 50 ng UM | 24 | p27 | 0 | 0 |
|  | 24 | p45 | 0 | 0 |
|  |  |  |  |  |
| 100 pg Chem | 10 | p27 | 10 | 100 |
|  | 10 | p45 | 10 | 100 |
|  |  |  |  |  |
| 50 pg Chem | 10 | p27 | 10 | 100 |
|  | 10 | p45 | 10 | 100 |
|  |  |  |  |  |
| 25 pg Chem | 24 | p27 | 24 | 100 |
|  | 24 | p45 | 24 | 100 |
|  |  |  |  |  |
| 12.5 pg Chem | 24 | p27 | 20 | 83.3 |
|  | 24 | p45 | 20 | 83.3 |
|  |  |  |  |  |
| 6.25 pg Chem | 24 | p27 | 14 | 58.3 |
|  | 24 | p45 | 14 | 58.3 |
